# Chelating-agent-assisted control of CsPbBr_3_ quantum well growth enables stable blue perovskite emitters

**DOI:** 10.1038/s41467-020-17482-0

**Published:** 2020-07-22

**Authors:** Ya-Kun Wang, Dongxin Ma, Fanglong Yuan, Kamalpreet Singh, Joao M. Pina, Andrew Johnston, Yitong Dong, Chun Zhou, Bin Chen, Bin Sun, Hinako Ebe, James Fan, Meng-Jia Sun, Yuan Gao, Zheng-Hong Lu, Oleksandr Voznyy, Liang-Sheng Liao, Edward H. Sargent

**Affiliations:** 1 Department of Electrical and Computer Engineering, University of Toronto, 10 King’s College Road, Toronto, Ontario M5S 3G4 Canada; 2 Institute of Functional Nano and Soft Materials (FUNSOM), Jiangsu Key Laboratory for Carbon-Based Functional Materials and Devices, Soochow University, 215123 Suzhou, Jiangsu PR China; 3 Department of Materials Science and Engineering, University of Toronto, 184 College Street, Toronto, Ontario M5S 3G4 Canada; 4 Department of Physical and Environmental Sciences, University of Toronto Scarborough, 1065 Military Trail, Scarborough, Ontario M1C 1A4 Canada

**Keywords:** Nanoscale materials, Nanoscale materials, Electronic devices, Electronic devices

## Abstract

Metal halide perovskites have emerged as promising candidates for solution-processed blue light-emitting diodes (LEDs). However, halide phase segregation – and the resultant spectral shift – at LED operating voltages hinders their application. Here we report true-blue LEDs employing quasi-two-dimensional cesium lead bromide with a narrow size distribution of quantum wells, achieved through the incorporation of a chelating additive. Ultrafast transient absorption spectroscopy measurements reveal that the chelating agent helps to control the quantum well thickness distribution. Density functional theory calculations show that the chelating molecule destabilizes the lead species on the quantum well surface and that this in turn suppresses the growth of thicker quantum wells. Treatment with γ-aminobutyric acid passivates electronic traps and enables films to withstand 100 °C for 24 h without changes to their emission spectrum. LEDs incorporating γ-aminobutyric acid-treated perovskites exhibit blue emission with Commission Internationale de l'Éclairage coordinates of (0.12, 0.14) at an external quantum efficiency of 6.3%.

## Abstract (web summary)

Metal halide perovskites have been studied as promising materials for blue light-emitting diodes (LEDs) but the stability is still a bottleneck. Here Wang et al. develop a chelating additive strategy to increase efficiency, operational stability and color stability of blue perovskite LEDs.

## Introduction

Metal halide perovskites are promising materials for light-emitting diodes (LEDs) due to their high photoluminescence quantum yields, narrow emission linewidths and defect tolerance^1–6^. Perovskites are tuned across a broad spectral range via chemical composition, particularly through partial halide substitution^7^. Partial substitution of Br and Cl results in the bandgap that achieves blue emission at the desired CIE coordinates (CIEy < 0.16). However, mixed Cl/Br perovskites undergo halogen phase segregation during LED operation, which leads to a shift in the emission spectrum away from the desired wavelength^8–10^.

Perovskite bandgaps can also be tuned through quantum confinement^7,11^. Low-dimensional analogs of 3D perovskites are obtained by inserting large organic ligands that divide the lattice into a finite number (*n*) of inorganic monolayers. Increasing the organic ligand content decreases the average *n* value and increases the bandgap^12,13^. Extra ligands (e.g., IPABr or PEABr) have been applied to achieve blue emission, but limited control over the distribution of quantum well (QW) widths results in sky-blue emission (longer than 490 nm) and low device performance (EQE below 1.5%). Other strategies in controlling QW thickness also lead to wide *n* distributions: these films exhibit either redshift of the TA signal wavelength or asymmetric emission peaks^14,15^. There is, as a result, significant interest in controlling the QW distribution while keeping photoluminescence quantum yield high.

The dynamic nature of QW formation produces a distribution of differently-sized QWs with various bandgaps^16^. The thin (low-*n*) QWs funnel high-energy excitons to the lowest-bandgap (high-*n*) perovskite QWs, shifting the emission profile to green, even when the concentration of larger-*n* QWs is low. It is thus critical to suppress *n* > 3 QW formation to ensure the emission remains blue^17,18^. Previous reports of bromide-containing perovskites have revealed thermal instability: small-*n* QWs form during the spin-coating process but, grow into larger ones upon annealing (at 90 °C within 1 min), shifting emission to green^12^.

We reasoned that a chemical strategy that controls the dynamics of CsPbBr_3_ QW growth—both limiting the *n* values formed during processing, and then stabilizing them—could produce blue emission from reduced-dimensional perovskites that is both efficient and stable. We employed a chelating amino acid molecule, γ-aminobutyric acid (GABA). We hypothesized that lead bromide molecules could be coordinated by GABA, improving their solubility, and thereby inhibiting the growth of thicker perovskite layers. In addition, GABA, a zwitterion, should passivate undercoordinated Pb sites that are otherwise susceptible to a reaction with oxygen.

By adding GABA, we achieve stable and tunable blue emission in the 464–475 nm range. GABA-treated CsPbBr_3_ QWs exhibit good thermal and emission stability with no spectral shift following 24 h annealing at 100 °C. Levering the improved emission, we fabricate spectrally stable (under operating biases as high as 12 V) blue LEDs that achieve external quantum efficiency of 6.3% with CIE coordinate of (0.12, 0.14).

## Results

### Quantum well size distribution analysis

**Fig. 1:**
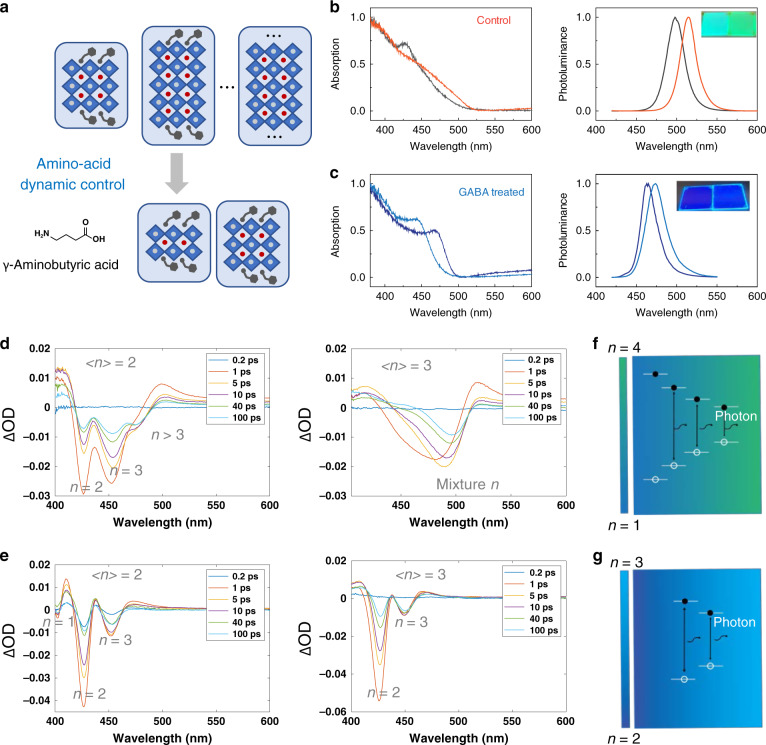
Photoexcited carrier dynamics of GABA-treated CsPbBr_3_ QWs. **a** Schematics depicting suppression by GABA of *n* < 2 and *n* > 3 QWs. **b**, **c** Absorption and photoluminescence spectra of the control (**b**) and GABA-treated (**c**) CsPbBr_3_ thin films. Inset: photograph of the perovskite films under external photoexcitation. **d**, **e** TAS of control (**d**) and GABA-treated (**e**) CsPbBr_3_ QWs with <*n*> = 2 and 3 at different delay times. **f**, **g** Schematics of photocarrier dynamics and recombination of the control (**f**) and GABA-treated (**g**) CsPbBr_3_ thin films.

We first examined the effects of incorporating GABA on *n* QW distribution with absorption and photoluminescence spectra (PL) measurements. Absorption of the control CsPbBr_3_ films—made with a precursor stoichiometry of <*n*> = 2 (<*n*> will henceforth refer to the precursor stoichiometry, while *n* will refer to a specific QW)—exhibited an excitonic absorption peak at 425 nm, and had an emission peak at 495 nm (Fig. 1a, b). Adjusting the ligand stoichiometry in the precursor solution to <*n*> = 3 redshifts the emission to around 510 nm while absorption becomes broad and featureless. The large Stokes shift indicates that there is a small concentration of larger *n* QWs into which created excitons are funneled. In contrast, GABA-treated CsPbBr_3_ thin films of <*n*> = 2 and 3 both show an excitonic peak at around 450 nm and exhibit deep-blue PL emission centered at 464 and 475 nm with CIE coordinates of (0.13, 0.07) and (0.11, 0.13), respectively (Fig. 1c and Supplementary Fig. 1). This indicates that the formation of larger *n* QWs is suppressed with the inclusion of GABA.

We sought further insight into the dynamics of photocarriers in both GABA-treated and pristine thin films. We employed femtosecond transient absorption spectroscopy (TAS) to probe the exciton dynamics at ultrafast timescales (Fig. 1d, e). Control CsPbBr_3_ thin films (<*n*> = 2 precursor solution) initially exhibit two distinctive bleach peaks at 430 nm and 450 nm corresponding to *n* = 2 and 3 (Fig. 1d). The two bleach peaks show a slight redshift (around 2 nm), commensurate with the appearance of an *n* > 3 shoulder bleach peak at a longer wavelength (around 480 nm) after 1 ps. <*n*> = 3 films show a broad bleach signal that shifts to longer wavelengths as the delay time is increased (Fig. 1d).

In contrast, TAS for GABA-treated CsPbBr_3_ thin films (<*n*> = 2) shows bleach peaks at 405, 430, and 450 nm, corresponding to *n* = 1, 2, 3, whose features are stable within the 100 ps timeframe (Fig. 1e). The TAS of larger CsPbBr_3_ QWs (<*n*> = 3) show no bleach peak at 405 nm, while the remaining bleach peaks are similar. For both <*n*> = 2 and <*n*> = 3 thin films, the GABA-treated films exhibit a narrower and more stable distribution of QWs.

### Controlling quantum well size distribution with a chelating agent

**Fig. 2:**
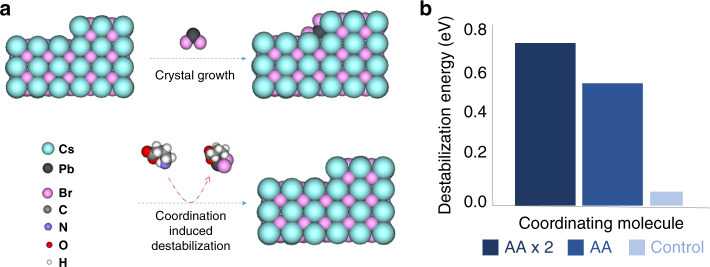
Control of 2D perovskite crystallization using a chelating agent. **a** Schematic of the chelating effect on PbBr_2_ binding to the surface. **b** DFT-calculated destabilization energy of PbBr_2_ on the surface of perovskite when coordinated with 2 GABA molecules, 1 GABA molecule, and PEA.

We used density functional theory (DFT) calculations to explore the effect of incorporating GABA on the final QW distribution (Fig. 2). The calculations show that the coordination environment of PbBr_2_ is a key factor in the growth of the perovskite structures (Fig. 2a). The propensity of PbBr_2_ to be coordinated by small chelating molecules in the vicinity of perovskite can inhibit the binding of PbBr_2_ to the surface of the perovskite, suppressing the growth of larger-*n* crystals. As a control, we studied coordination by phenethylamine (PEA) molecules, finding it to result in a PbBr_2_ destabilization energy of 0.05 eV. Replacing the unidentate groups with a bidentate small molecule (GABA) resulted in a 10-fold increase in destabilization energy (0.51 eV) (Fig. 2b). This could be further increased by utilizing two GABA molecules for coordination. The 10-fold increase in destabilization energy helps to control the QW size distribution: for example, step edges are the key sites for deciding between growing vs. dissolving the next *n* layer; they are the most undercoordinated sites and thus are most active toward binding the precursors but are also the most susceptible sites for a chelating agent attack. Undercoordinated sites at step edges are also the primary sites for defect formation and eliminating them helps decrease the number of non-radiative recombination centers. To confirm the chelating effect, we tried two different amino acid ligands; 5-aminopetanoic acid and phenylalanine. Both amino acid-treated films show blue-shift PL spectra compared with the control films, indicating the general nature of this strategy (Supplementary Fig. 2).

### Structural and photophysical analysis

**Fig. 3:**
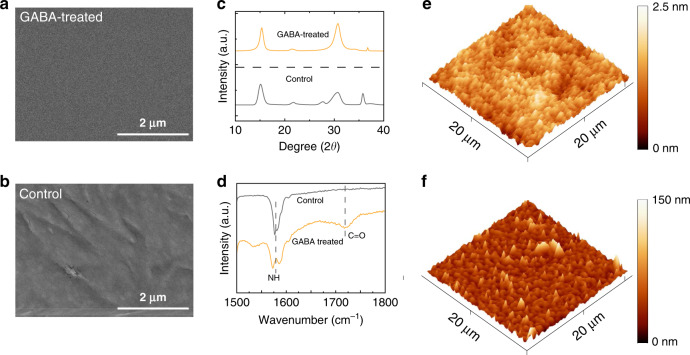
Structural and morphological properties of CsPbBr_3_ QW perovskites. **a**, **b** Top-view scanning electron microscope (SEM) images of GABA-treated (**a**) and control (**b**) CsPbBr_3_ QW films. **c** Powder X-ray diffraction (PXRD) of control and GABA-treated CsPbBr_3_ thin films. **d** FTIR spectra of control and GABA-treated CsPbBr_3_ QW films. **e**, **f** Atomic force microscope (AFM) images of GABA-treated (**e**) and control (**f**) CsPbBr_3_ thin films.

We used scanning electron microscopy (SEM) and atomic force microscopy (AFM) to investigate the film morphology of the control and GABA-treated thin films. SEM images of GABA-treated CsPbBr_3_ thin films show a flat and continuous film over a 2 μm area (Fig. 3a). In contrast, the control CsPbBr_3_ QWs shows a rougher film (Fig. 3b). Atomic force microscopy (AFM) images of GABA-treated CsPbBr_3_ QWs show a smoother morphology with an RMS of around 0.3 nm, which is much lower than that of control film (13.1 nm) (Fig. 3e, f). The better morphology of GABA-treated film is linked to improved control of crystallization and QW size distribution, as evidenced by TAS^19^. Powder X-ray diffraction (PXRD) of control and GABA-treated films show typical perovskite diffraction signals (Fig. 3c). Fourier transform infrared spectroscopy (FTIR) spectra of GABA-treated CsPbBr_3_ QWs show clear C=O absorption features at a 1500–1700 cm^−1^, which confirms that GABA is incorporated in the films, while pristine films are featureless in this range (Fig. 3d).

**Fig. 4:**
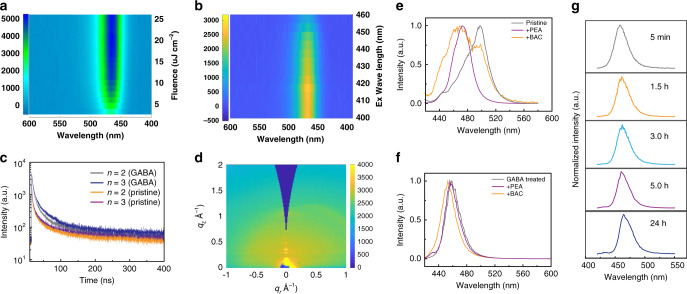
Optical characteristics of GABA-treated CsPbBr_3_ QWs. **a** Power-dependent photoluminescence spectra of GABA-treated CsPbBr_3_ thin films. **b** Excitation wavelength-dependent photoluminescence spectra of GABA-treated CsPbBr_3_ thin films. **c** Time-resolved photoluminescence (TRPL) decay of control and GABA-treated CsPbBr_3_ films. **d** GIWAXS pattern of CsPbBr_3_ thin films with GABA treatment. **e**, **f** Photoluminescence features under acidic (BAC) and basic (PEA) conditions for the control (**e**) and GABA-treated (**f**) CsPbBr_3_ QW films. **g** Thermal stability of GABA-treated CsPbBr_3_ QW films under 100 °C within the timeframe of 24 h.

Next we studied the power-dependent and excitation wavelength-dependent PL of the pristine and GABA-treated CsPbBr_3_ thin films (Fig. 4a, b). GABA-treated CsPbBr_3_ QWs exhibit an emission peak at 463 nm, which is 30 nm blue-shifted compared with the control sample (Fig. 4a and Supplementary Fig. 3a). The GABA-treated QWs exhibit no spectral shift with increasing excitation power, while control films show a 5 nm shift. PL excitation (PLE) spectra of pristine and GABA-treated CsPbBr_3_ QWs (<*n*> = 2) exhibit single emission peaks (463 and 495 nm for GABA-treated and control film, respectively) (Fig. 4b and Supplementary Fig. 3b). This suggests that the energy transfer from smaller to larger *n* QWs is efficient for both films.

Time-resolved photoluminescence (TRPL) decay of GABA-treated CsPbBr_3_ (<*n*> = 2 and 3) thin films show longer lifetimes of 13 and 15 ns, compared with 6 and 7 ns for control CsPbBr_3_ QWs (Fig. 4c). The longer PL lifetime of GABA-treated films suggests its passivation effects due to the Lewis base property of COO^−^ group, consistent with previous literature^20^. The longer lifetime of GABA-treated samples, taken with the higher PLQY (GABA-treated films of 82% and control of 40%), suggests that GABA passivates traps and reduces the non-radiative recombination rate in the films. We employed grazing-incidence wide-angle X-ray scattering (GIWAXS) to probe the orientation of thin films. The interlayer spacing between 2D perovskite layers (*n* = 1) is around 17 Å, and the corresponding diffraction peak is at 0.37 Å^−1 ^^14^. Diffraction peaks observed at smaller *q* values are the result of higher (*n* = 2 and 3) *n* domains. These diffraction peaks are primarily observed as spots (instead of rings) along the *q*_z_ axis. From this, we conclude that the quasi-2D perovskite layers are aligned parallel to the substrate.

### Emission stability

We further investigated materials and emission stability under heat/acid/base conditions of GABA-treated CsPbBr_3_ films and control films. We choose benzoic acid (BAC) and phenethylamine (PEA) as acid and base sources to investigate CsPbBr_3_ QWs emission stability. Untreated CsPbBr_3_ QWs show a broadening of the emission after both treatments, suggesting that the QW distribution is unstable under these conditions (Fig. 4e), and consistent with DFT results that a chelating agent can delaminate the QWs layer by layer. However, the chelating strength of PEA and BAC is not sufficient to completely suppress *n* = 4 layers, resulting in a broader thickness distribution. In contrast, the emission of GABA-treated CsPbBr_3_ QWs is largely unchanged after acid and base treatment, from which we conclude that the GABA-treated films are stable under both conditions (Fig. 4f). Similarly, GABA-treated CsPbBr_3_ QWs are also stable after heating at 100 °C for 24 h (Fig. 4g).

### Device performance and operational stability

**Fig. 5:**
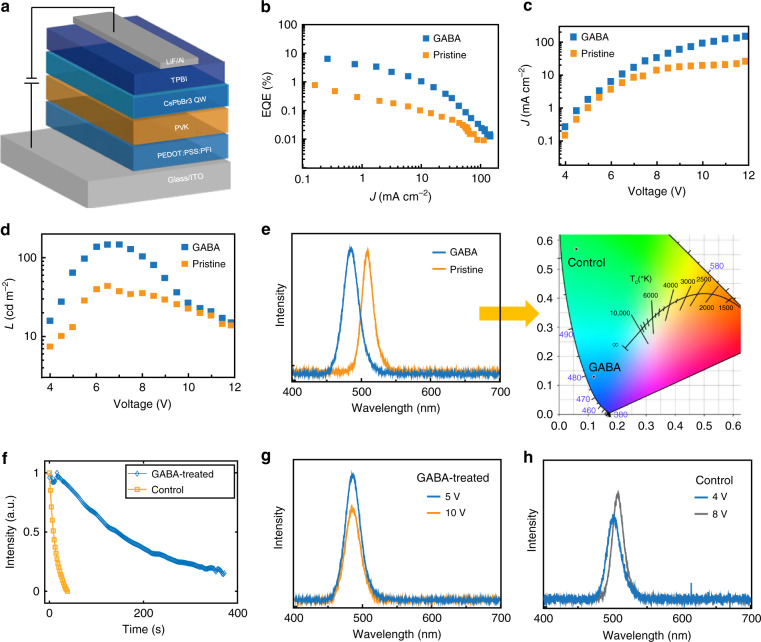
CsPbBr_3_ QW LED performance. **a** Schematic of LEDs fabricated using CsPbBr_3_ perovskite QW films. **b**, **c**, **d** EQE–*J*, *J–V* and *L–V* curves of LED devices. **e** Electroluminescence (EL) spectra of GABA-treated CsPbBr_3_ QWs and control QW film LEDs and corresponding CIE coordinates. **f** Operational stability of GABA-treated and control LEDs at maximum luminescence. **g**, **h** EL spectra of GABA-treated and control devices at different voltages.

Levering the improved stability and PLQYs, we incorporated the GABA-treated CsPbBr_3_ QWs in LEDs. We use an ITO glass substrate anode, a poly(3,4-ethylenedioxythiophene): poly(styrene-sulfonate) (PEDOT:PSS) mixed with Nafion hole injection layer (HIL), a poly(9-vinylcarbazole) (PVK) hole transport layer (HTL), a CsPbBr_3_ QW emission layer, a 1,3,5-tris(N-phenylbenzimidazol-2-yl) benzene (TPBI) electron transport layer (ETL), and a LiF/Al double-layered cathode, as shown in Fig. 5a and Supplementary Fig. 4. The CsPbBr_3_ QWs with GABA treatment exhibit true-blue emission (478 nm) with CIE coordinates of (0.12, 0.14), whereas untreated devices emit at green wavelengths with CIE coordinates of (0.06, 0.57) (Fig. 5e).

Luminescence (*L*) and current density (*J*) curves as a function of voltage (*V*) for control and GABA-treated devices are shown in Fig. 5b–d. The maximum luminescence of GABA-treated LEDs is around 200 cd m^−2^, which is more than 2× higher than the pristine devices (Fig. 5d). The GABA-treated CsPbBr_3_ QW LEDs exhibit a maximum EQE of 6.3% (at 4 V, 0.26 mA, and 17 cd m^−2^) (Fig. 5a and Supplementary Fig. 5), which is among the best reported blue perovskite LEDs^13,17,18,21–24^. We attribute the EQE and luminescence to the improved passivation of uncoordinated Pb ions by GABA.

We investigated the device stability of GABA-treated and control films under different operating conditions. The GABA-treated blue LEDs have a T50 of 150 s at 200 cd m^−2^, which is an order of magnitude larger than the control device (Fig. 5f). To determine if the improved device stability was a result of the improved phase (and thermal) stability of the QW distribution, we check the EL spectrum under operating conditions. GABA-treated devices maintain consistent EL emission under different voltages (up to a bias of 12 V) and after stability tests (Fig. 5g and Supplementary Fig. 6), while the EL of the untreated films shifted by 4 nm when the operating voltage reached to 8 V (Fig. 5h). The improved thermal stability of GABA-treated films inhibits the thermal degradation of the LEDs induced by device operation^25^.

## Discussion

In conclusion, we have demonstrated the use of chelating agents to increase PLQY and to control CsPbBr_3_ QW distribution. We incorporate γ-aminobutyric acid in the precursor solutions to passivate surfaces and destabilize the Pb sites to control the quantum well thickness distribution. Two additional amino acid ligands also blue-shift PL spectra compared with control films. We fabricated a true-blue LED with CIE coordinates of (0.12, 0.14) and an EQE of 6.3%. This strategy contributes in the direction of more efficient, spectrally stable, blue LEDs.

## Methods

### Materials and chemicals

Methylammonium bromide (MABr) and phenylethylammonium bromide (PEABr) were purchased from Dyesol. Lead bromide (PbBr_2_, 99.998%) and cesium bromide (CsBr, 99.999%) were purchased from Alfa Aesar. Anhydrous dimethyl sulfoxide (DMSO), anhydrous chloroform, Nafion perfluorinated resin solution (tetrafluoroethylene-perfluoro-3,6-dioxa-4-methyl-7-octenesulfonic acid copolymer, 5 wt% in a mixture of lower aliphatic alcohols and water, containing 45% water), and lithium fluoride (LiF, >99.99%) were purchased from Sigma-Aldrich. Poly(3,4-ethylenedioxythiophene)polystyrene sulfonate (PEDOT:PSS, CleviosTM PVP Al 4083) was purchased from Heraeus. 1,3,5-tris(N-phenylbenzimiazole-2-yl)benzene (TPBi) was purchased from Lumtec. All the chemicals were used directly as received.

### Perovskite film fabrication

The precursor solution is prepared with equation of PEA_2_Cs_n−1_Pb_n_Br3_n+1_. For GABA-treated solution, different molar ratio of GABA (0.05 and 0.1 M) is added in the precursor solution in DMSO under continuous stirring for 30 min in a nitrogen-filled glovebox at room temperature. The precursor was spin-coated onto a glass substrate using a two-step method. The resulted clear and colorless solution was dripped onto the substrates after filtration, pre-spun at 1000 rpm for 10 s, then spin-coated at 5000 rpm for 60 s. After 25 s during the second step, 400 µL of chloroform dissolving TPPO (98%, Sigma-Aldrich) is deposited onto the perovskite film during the second step. Triphenylphosphine oxide (TPPO) is a passivating agent that has been reported in previous literature^26^. The second step is key to the crystal growth process as this step utilizes the antisolvent to form better film and control the perovskite growth^27^. The resulting films were then annealed at 90 °C for 5 min to increase crystallization. The 0.05 M for <*n*> = 3 is the best concentration for fabricating LEDs.

### DFT calculations

Calculations were performed using the CP2K computational package with the Quickstep module^28^. The calculations employed a grid cut-off of 600 Ry, Goedecker–Teter–Hutter pseudopotentials^29^, Perdew–Burke–Ernzerhof exchange-correlation functional^30^ and MOLTOPT double-zeta plus polarized orbital basis sets. The slabs were separated by 55 Å of vacuum in the *y*-direction and made periodic in the *x* and *z* direction. All geometries were relaxed until forces on atoms converged to below 40 meV Å^−1^. The destabilization energy was calculated as the difference in energies of the structure with molecule-coordinated PbBr_2_ and the structure with PbBr_2_ adsorbed on surface and non-coordinating molecules present within the same surface unit cell:

1 $$E_{{\mathrm{destabilization}}} = E_{{\mathrm{non-coordinated}}} - E_{{\mathrm{coordinated}}}$$

### Absorption, PL, PL lifetime, and PLQY measurements

Absorption spectra for CsPbBr_3_ QWs were collected using a fiber coupled modular spectrometer (USB 2000+, ocean optics). PL spectra of CsPbBr_3_ QWs thin films were collected using a Horiba Fluorolog system with a xenon lamp as the excitation source. The sample was placed at an incidence angle of 30°. The PL spectra were collected using a calibrated monochromator:single-photon-detector assembly. The PL lifetime data was recorded on using a time-correlated single-photon counting (TCSPC) system (Horiba). PLQY is measured using an integrating sphere.

### Transient absorption (TA) measurements

TA spectra were recorded using a femtosecond (fs) pump–probe spectroscopy setup. The fs laser pulses were produced by a regeneratively amplified Yb:KGW laser (Light Conversion, Pharos) at 1 kHz repetition rate. The fundamental beam was split into two beams, with one passing through an optical parametric amplifier (OPA, Light Conversion Orpheus) to generate a pump pulse at 450 nm, and was chopped. The other portion of the beam was directed onto a CaF crystal after focusing to generate white-light supercontinuum as the probe light. The power of pump light was monitored using a power meter (Ophir) to keep excitation fluence lower than the Auger threshold. The time delay was adjusted using a translation stage, optically delaying the probe pulses. The probe light intensity was measured with a CCD detector. The sample was translated at a speed of 1 mm/s during measurements. TA bleach recovery dynamics were recorded at the bandedge bleach peak position.

### X-ray scattering measurements

Powder X-ray diffraction pattern was obtained using Rigaku Miniflex 600 6G benchtop powder X-ray diffraction instrument with a Cu K X-ray source. The CsPbBr_3_ QWs films were prepared on a glass substrate.

Grazing-incidence wide-angle X-ray spectroscopy (GIWAXS) was conducted at the Hard X-ray MicroAnalysis (HXMA) beamline of the Canadian Light Source (CLS). The energy of 17.998 keV (*λ* = 0.6888 Å) was selected using a Si (111) monochromator. Patterns were collected on a SX165 CCD camera (Rayonix) placed at 175 mm from the sample. A lead beamstop blocked the direct beam. Images were calibrated using LaB_6_ and processed with the Nika Igor plug-in and the GIXSGUI MATLAB plug-in.

### AFM and SEM measurements

AFM measurements were performed with an Asylum Research Cypher AFM operated in AC mode in air. Imaging was done using ASYELEC-02 silicon probes with titanium–iridium coatings from Asylum Research. The probes had a typical spring constant of 42 N m^−1^. SEM images were collected in secondary electron mode by a Hitachi SU5000. The measurements were operated at a voltage of 3 kV with a spot size of 3 and an intensity of 10.

### FTIR

spectra were collected using a Thermo Scientific Nicolet iS50 ATR-FTIR system. Spectra were obtained using 16 scans with a resolution of 4 cm^−1^. All CsPbBr_3_ QWs thin film samples were spin-coating on glass substrates.

### LED fabrication

First the low-conductivity ITO-coated glass substrates were sequentially cleaned by detergent, deionized water, acetone, and isopropanol in an ultrasonic washer, then treated by ultraviolet ozone plasma for 5 min and employed as the anode. Then a mixed solution of PEDOT:PSS:PFI (at the mass ratio of 4:1) was spin-coated at 2000 rpm for 20 s, followed by annealing on a hotplate at 150 °C for 20 min in the air ambient. After the substrate was cooled to room temperature, 6 mg ml^−1^ PVK chlorobenzene solution was spin-coated onto the PEDOT:PSS layer and bake for 15 min at 170 °C. Perovskite films were fabricated as described above. Finally, the substrates were transferred into a high vacuum thermal evaporator, where TPBi (60 nm), LiF (1 nm), and Al (150 nm) were deposited thereon layer by layer through a shadow mask under a high vacuum of <10^−4^ Pa. The device active area was 6.14 mm^2^ as defined by the overlapping area of the ITO and Al electrodes. The devices were encapsulated before the measurements, using an ultraviolet curable resin (exposure under ultraviolet light for 20 s) and covered on the edges between the device and a transparent glass chip. The LED stability was measured in a glovebox at room temperature under dark conditions. The PL spectra for stability were measured under ambient conditions with humidity of 30% and temperature 25 °C. We have reproduced the measurement for multiple and for several repetitions of the same experiment.

### LED evaluation

All devices were tested under ambient conditions. The luminance versus voltages and the current density versus voltage characteristics were collected using a HP4140B picoammeter. The absolute EL power spectra were collected using an integrating sphere and an Ocean Optics USB4000 spectrometer by mounting of the devices on the wall of the integrating sphere. The EQEs were then calculated through the measured absolute EL power spectra and the current density.

## Supplementary information

Supplementary Information

## Data Availability

The data that support the findings of this study are available from the corresponding author upon reasonable request.

## References

[1] Tan Z-K (2014). Bright light-emitting diodes based on organometal halide perovskite. Nat. Nanotechnol..

[2] Wang J (2015). Interfacial control toward efficient and low-voltage perovskite light-emitting diodes. Adv. Mater..

[3] Lin K (2018). Perovskite light-emitting diodes with external quantum efficiency exceeding 20 per cent. Nature.

[4] MacLaughlin CM (2019). Opportunities and challenges in perovskite-based display technologies: a conversation with Andrey Rogach and Haibo Zeng. ACS Energy Lett..

[5] Chen Q (2014). Controllable self-Induced passivation of hybrid lead iodide perovskites toward high performance solar cells. Nano. Lett..

[6] Cho H (2015). Overcoming the electroluminescence efficiency limitations of perovskite light-emitting diodes. Science.

[7] Protesescu L (2015). Nanocrystals of cesium lead halide perovskites (CsPbX3, X = Cl, Br, and I): Novel optoelectronic materials showing bright emission with wide color gamut. Nano Lett..

[8] Chiba T (2018). Anion-exchange red perovskite quantum dots with ammonium iodine salts for highly efficient light-emitting devices. Nat. Photonics.

[9] Zhang H (2019). Phase segregation due to ion migration in all-inorganic mixed-halide perovskite nanocrystals. Nat. Commun..

[10] Li G (2016). Highly efficient perovskite nanocrystal light-emitting diodes enabled by a universal crosslinking method. Adv. Mater..

[11] Tyagi P, Arveson SM, Tisdale WA (2015). Colloidal organohalide perovskite nanoplatelets exhibiting quantum confinement. J. Phys. Chem. Lett..

[12] Xing J (2018). Color-stable highly luminescent sky-blue perovskite light-emitting diodes. Nat. Commun..

[13] Pan J (2018). Bidentate kigand-passivated CsPbI_3_ perovskite nanocrystals for stable near-unity photoluminescence quantum yield and efficient red light-emitting diodes. J. Am. Chem. Soc..

[14] Jin Y (2020). Synergistic effect of dual ligands on stable blue quasi-2D perovskite light-emitting diodes. Adv. Funct. Mater..

[15] Yantara N (2020). Designing the perovskite structural landscape for efficient blue emission. ACS Energy Lett..

[16] Quintero-Bermudez R (2018). Compositional and orientational control in metal halide perovskites of reduced dimensionality. Nat. Mater..

[17] Jiang Y (2019). Spectra stable blue perovskite light-emitting diodes. Nat. Commun..

[18] Liu Y (2019). Efficient blue light-emitting diodes based on quantum-confined bromide perovskite nanostructures. Nat. Photonics.

[19] Liang Q (2016). Enhancing the crystallization and optimizing the orientation of perovskite films via controlling nucleation dynamics. J. Mater. Chem. A.

[20] Yang S (2019). Tailoring passivation molecular structures for extremely small open-circuit voltage loss in perovskite solar cells. J. Am. Chem. Soc..

[21] Wang Q (2019). Efficient sky-blue perovskite light-emitting diodes via photoluminescence enhancement. Nat. Commun..

[22] Hou S (2018). Efficient blue and white perovskite light-emitting diodes via manganese doping. Joule.

[23] Kumawat NK (2019). Blue perovskite light-emitting diodes: progress, challenges and future directions. Nanoscale.

[24] Li Z (2019). Modulation of recombination zone position for quasi-two-dimensional blue perovskite light-emitting diodes with efficiency exceeding 5%. Nat. Commun..

[25] Lee, W. et al. Ultralow thermal conductivity in all-inorganic halide perovskites. *Proc. Natl Acad. Sci. USA* **114**, 8693–8697 (2017). 10.1073/pnas.1711744114 PMC5565476 28760988

[26] Quan L (2020). Edge stabilization in reduced-dimensional perovskites. Nat. Commun..

[27] Tavakoli MM (2019). Controllable perovskite crystallization via antisolvent technique using chloride additives for highly efficient planar perovskite solar cells. Adv. Energy Mater..

[28] VandeVondele J (2005). QUICKSTEP: fast and accurate density functional calculations using a mixed Gaussian and plane waves approach. Comput. Phys. Commun..

[29] Goedecker S, Teter M, Hutter J (1996). Separable dual-space Gaussian pseudopotentials. Phys. Rev. B.

[30] Perdew JP, Burke K, Ernzerhof M (1996). Generalized gradient approximation made simple. Phys. Rev. Lett..

