# Supplementary material for: Chelating-agent-assisted control of CsPbBr3 quantum well growth enables stable blue perovskite emitters
Source: Nat Commun. 2020 Jul 22;11:3674. doi: 10.1038/s41467-020-17482-0 (PMC7376073; doi:10.1038/s41467-020-17482-0)
Supplement: Supplementary file 1 — Supplementary Information [file 41467_2020_17482_MOESM1_ESM.pdf]

Supplementary Information for

Chelating-agent-assisted control of $CsPbBr_3$ quantum well growth enables stable blue perovskite emitters

Ya-Kun Wang *et al*.

Supplementary Figures

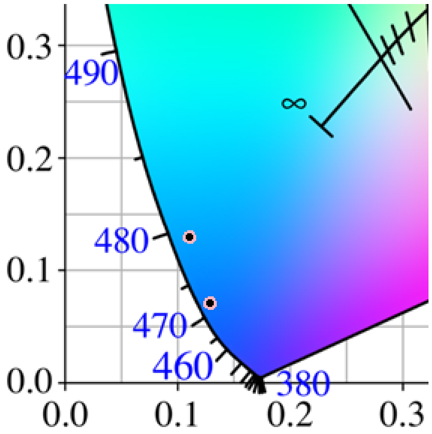

Supplementary Figure 1. CIE coordinate of <*n*> = 2 and 3 $CsPbBr_3$ thin-films with GABA-treatment.

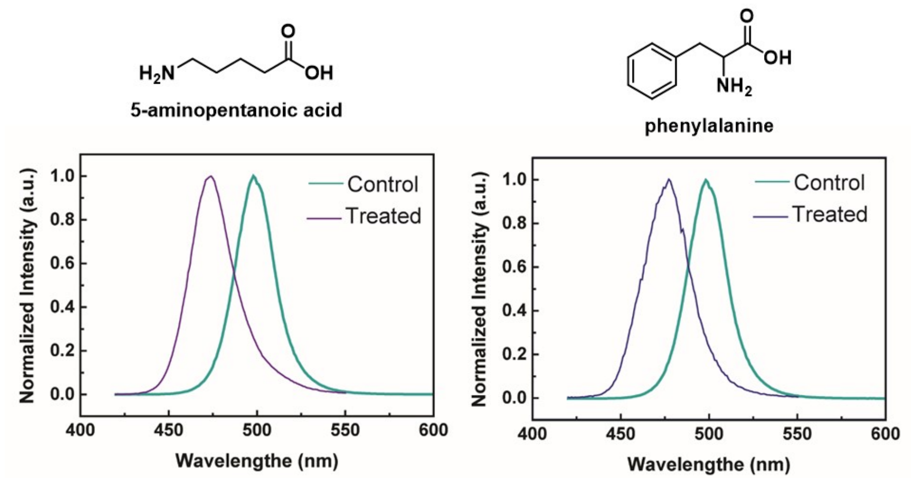

Supplementary Figure 2. PL spectra of control films other amino-acid-treated films.

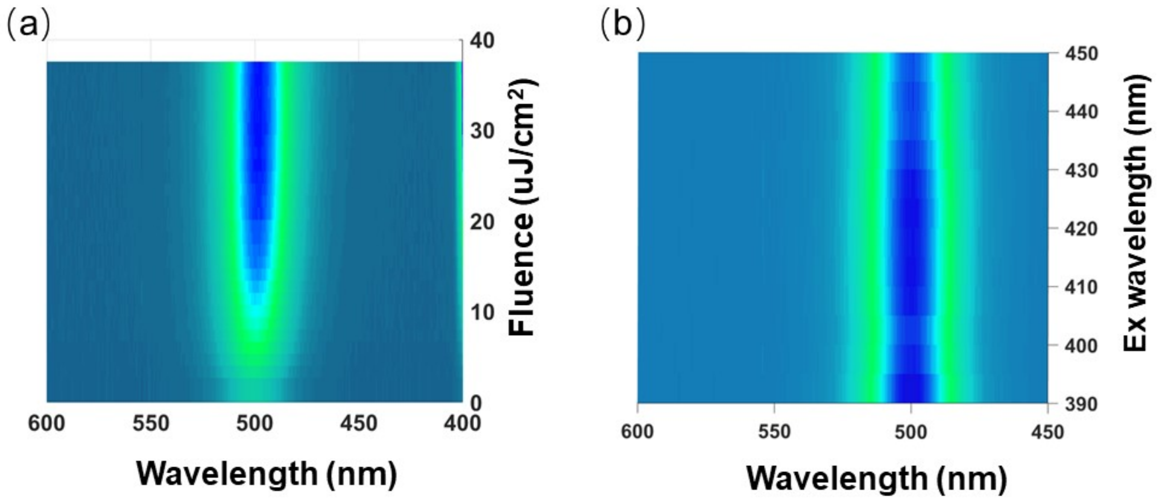

Supplementary Figure 3. (a) Power-dependent photoluminescence spectra and (b) excitation wavelength dependent photoluminescence spectra of control $CsPbBr_3$ thin-films.

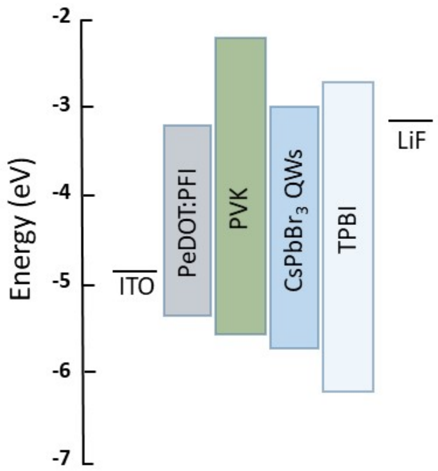

Supplementary Figure 4. Energy level of LEDs fabricated using perovskite QWs.

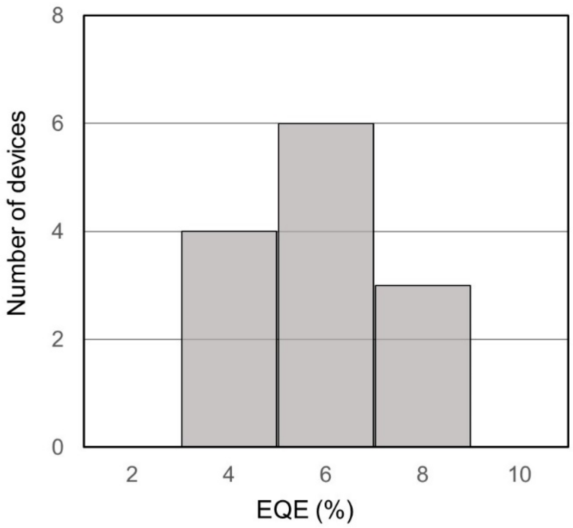

Supplementary Figure 5. EQE histograms of blue LED with GABA-treatment.

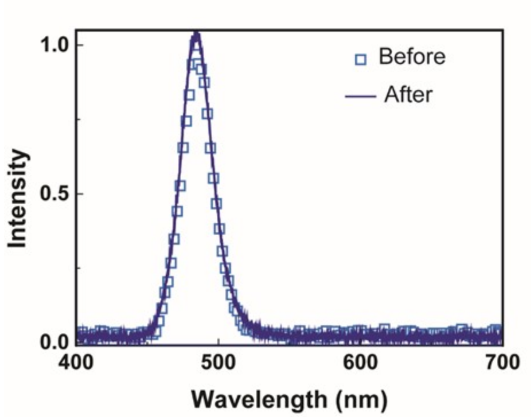

Supplementary Figure 6. EL spectra of GABA-treated $CsPbBr_3$ device before and after stability measurement.
